# Supplementary material for: PURE makes PURE: reconstitution of the PURE cell-free system from self-synthesized proteins
Source: Nat Commun. 2026 May 22;17:6756. doi: 10.1038/s41467-026-73337-0 (PMC13385756; doi:10.1038/s41467-026-73337-0)

Supporting Information

PURE makes PURE: reconstitution of the PURE cell-free system from self-synthesized proteins

Seyed Saeed Mottaghi[1] and Sebastian J. Maerkl[*1]

[1]Institute of Bioengineering, School of Engineering, École Polytechnique Fédérale de Lausanne, Lausanne, Switzerland
[*]*Correspondence: sebastian.maerkl@epfl.ch*

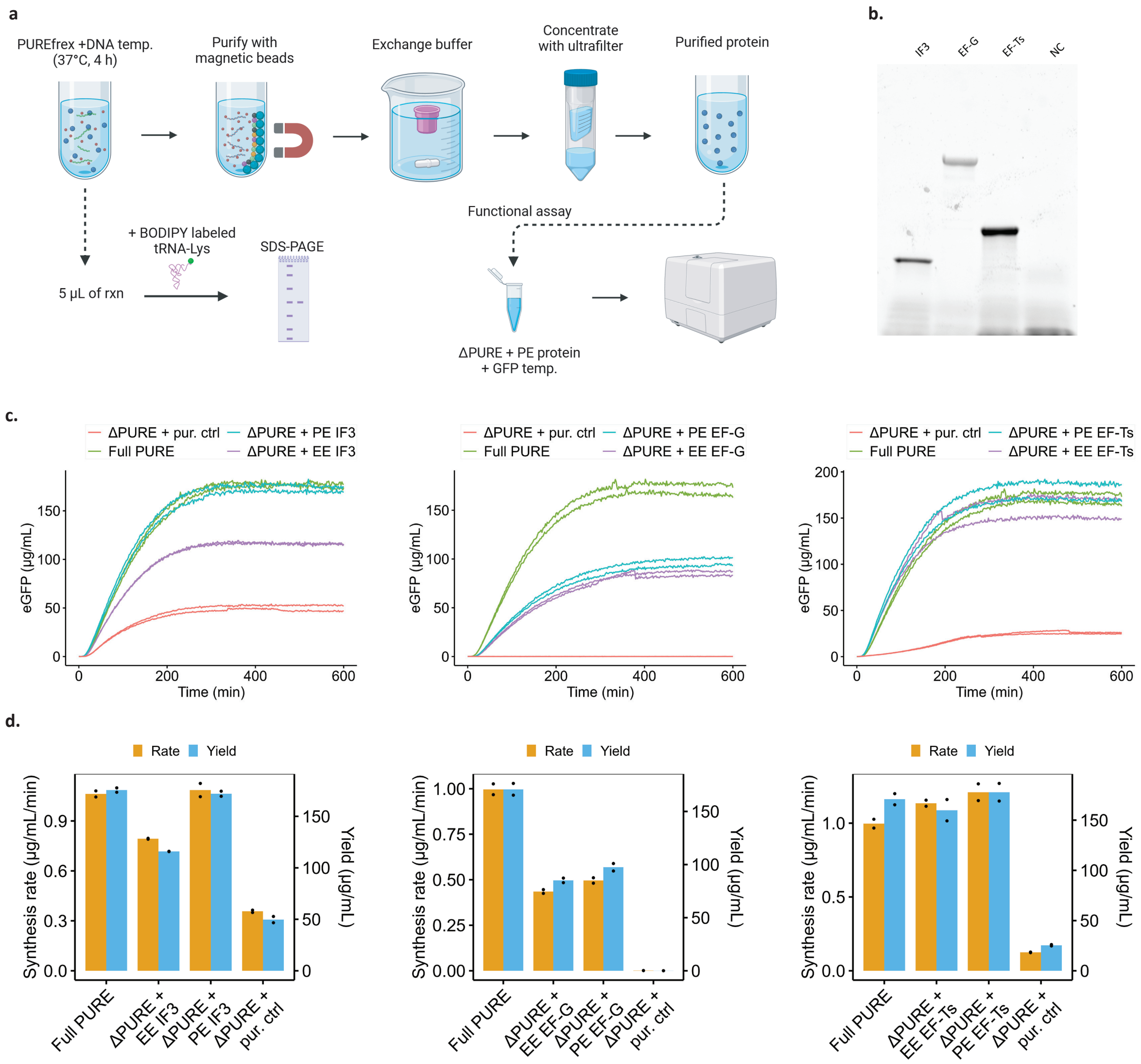

Supplementary Figure 1: **Functional evaluation of individual proteins from the TLF subset.** **a,** Workflow for the expression and purification of proteins using PUREfrex and their subsequent functional evaluation. Created in BioRender. Mottaghi, S. S. (2026) https://BioRender.com/hmbwy14. **b,** SDS–PAGE analysis of PUREfrex reactions expressing individual proteins. The gel displays the BODIPY-lysine fluorescence of each synthesized protein. Full uncropped scans of gels are appended to the end of the Supporting Information. **c,** Functional assay results for each protein. The plot represents eGFP expression level (μg/mL) for each reaction (n=2 for all conditions, technical replicates). Full PURE is included as the positive control, ΔPURE supplemented with a purification control as the negative control, and the adjusted control was prepared from *E. coli* expressed proteins at equivalent concentrations added to the ΔPURE reaction. **d,** Yield (μg/mL) and rate (μg/mL/min) of eGFP synthesis calculated from the data in panel **c**. Bars represent the average, and dots represent individual data points. EE: *E. coli* expressed, PE: PURE expressed, NC: negative control, pur. ctrl: purification control. Source data are provided as a Source Data file.

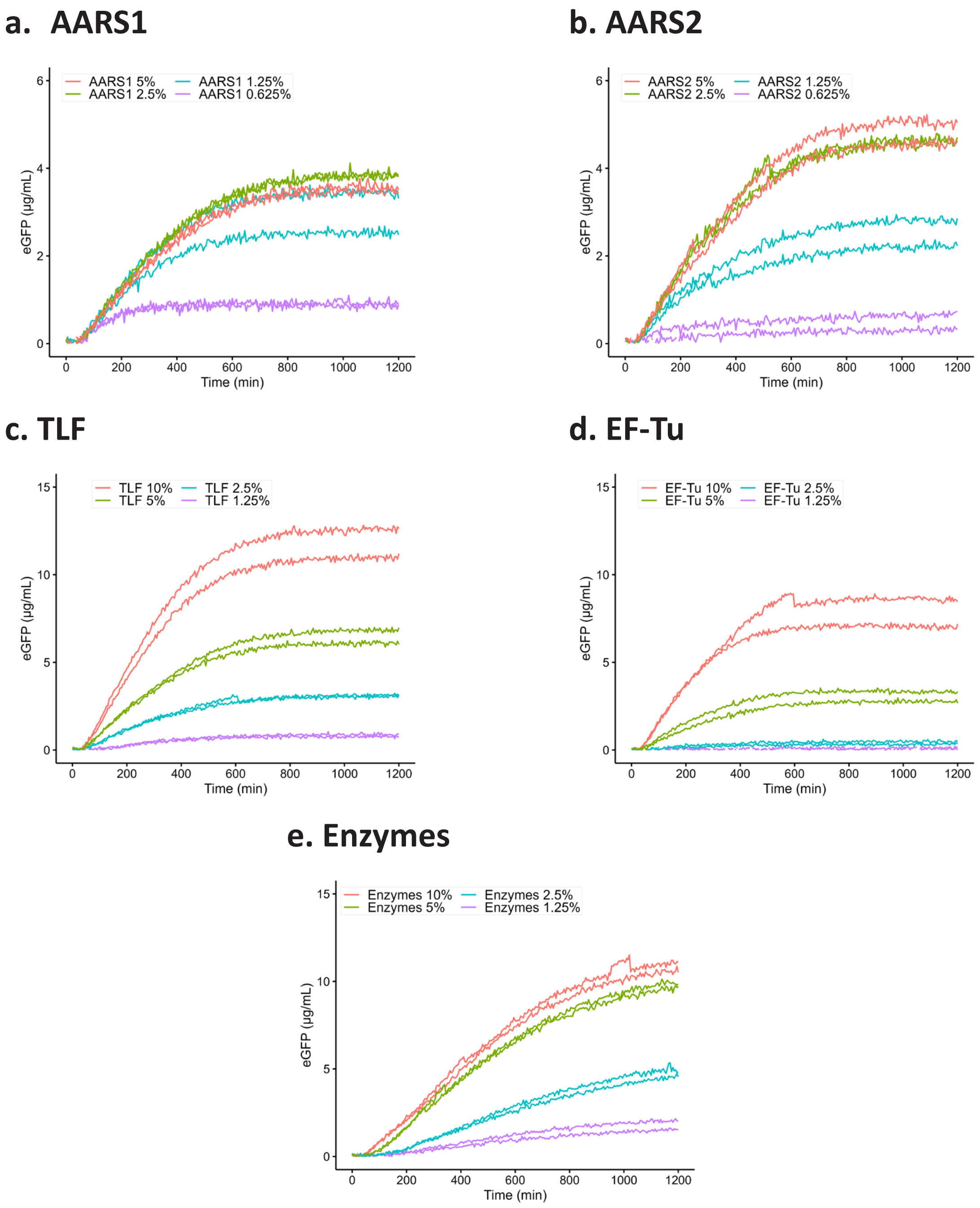

Supplementary Figure 2: **Titration plots of PURE expressed protein subsets.** (a–e) Serial dilutions of each subset were prepared and added to the corresponding 10-fold diluted ΔPURE reaction (containing a 10-fold lower concentration of non-ribosome proteins) containing an eGFP template, and fluorescence was measured over time. The plot shows the eGFP expression level (µg/mL) in each reaction (n=2 for all conditions, technical replicates). The rates in the titration plots in Figure 2a are based on these results. Source data are provided as a Source Data file.

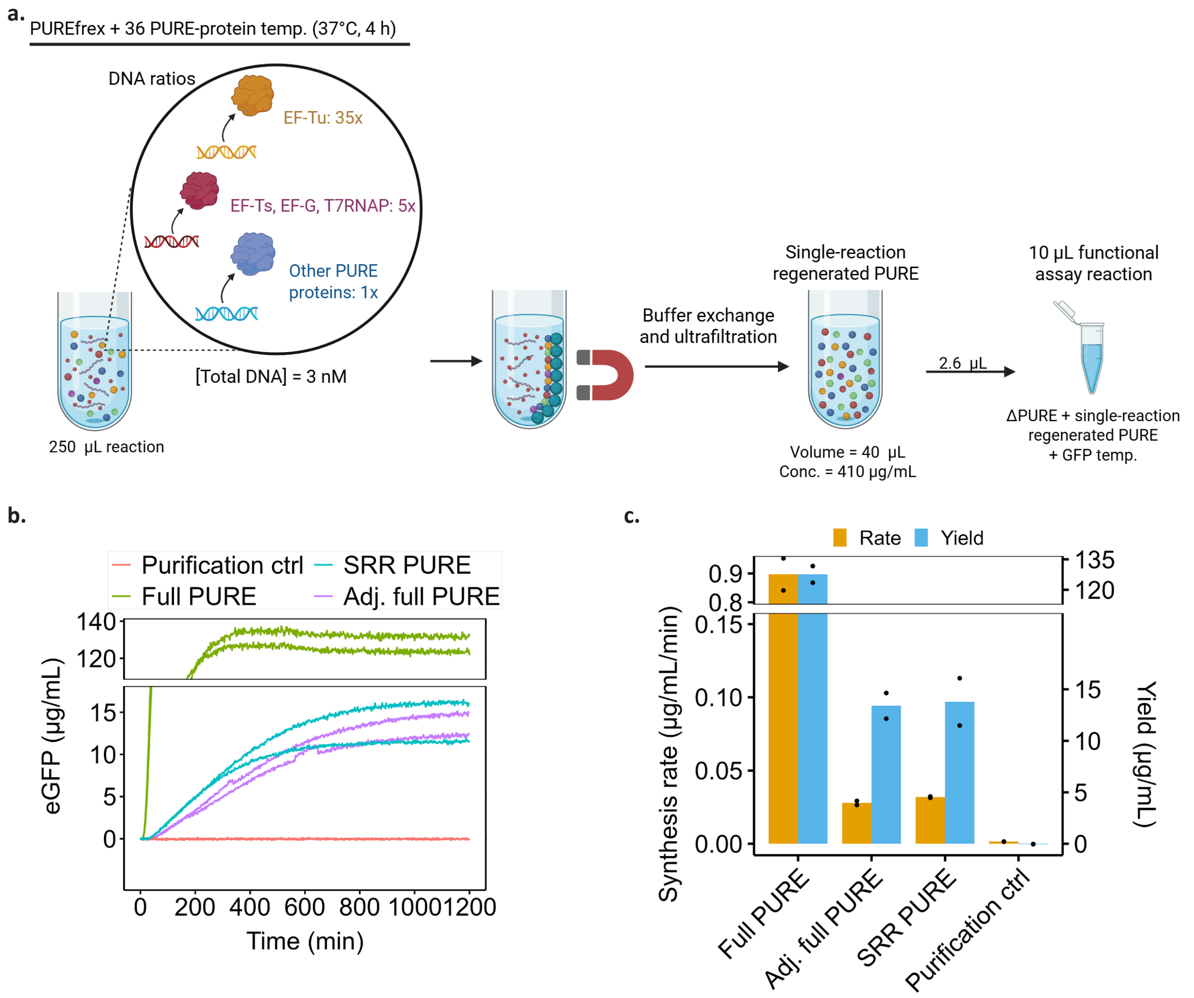

Supplementary Figure 3: **Repeat of single-reaction regeneration of PURE. a,** Repeat of single-reaction regeneration of PURE proteins in PURE. Created in BioRender. Mottaghi, S. S. (2026) https://BioRender.com/0kyzplv. **b,** Functional assay result of single-reaction regenerated PURE. The plot shows the eGFP expression level (μg/mL) in each reaction (n=2 for all conditions, technical replicates). Full PURE is included as the positive control, ΔPURE supplemented with a purification control as the negative control, and the adjusted control was prepared from homemade full PURE diluted to the same final total concentration and added to the ΔPURE reaction. **c,** Yield (μg/mL) and rate (μg/mL/min) of eGFP synthesis calculated from panel **b**. Bars represent the average, and dots represent individual data points. Adj. full PURE: concentration adjusted full PURE, SRR PURE: single-reaction regenerated PURE. Source data are provided as a Source Data file.

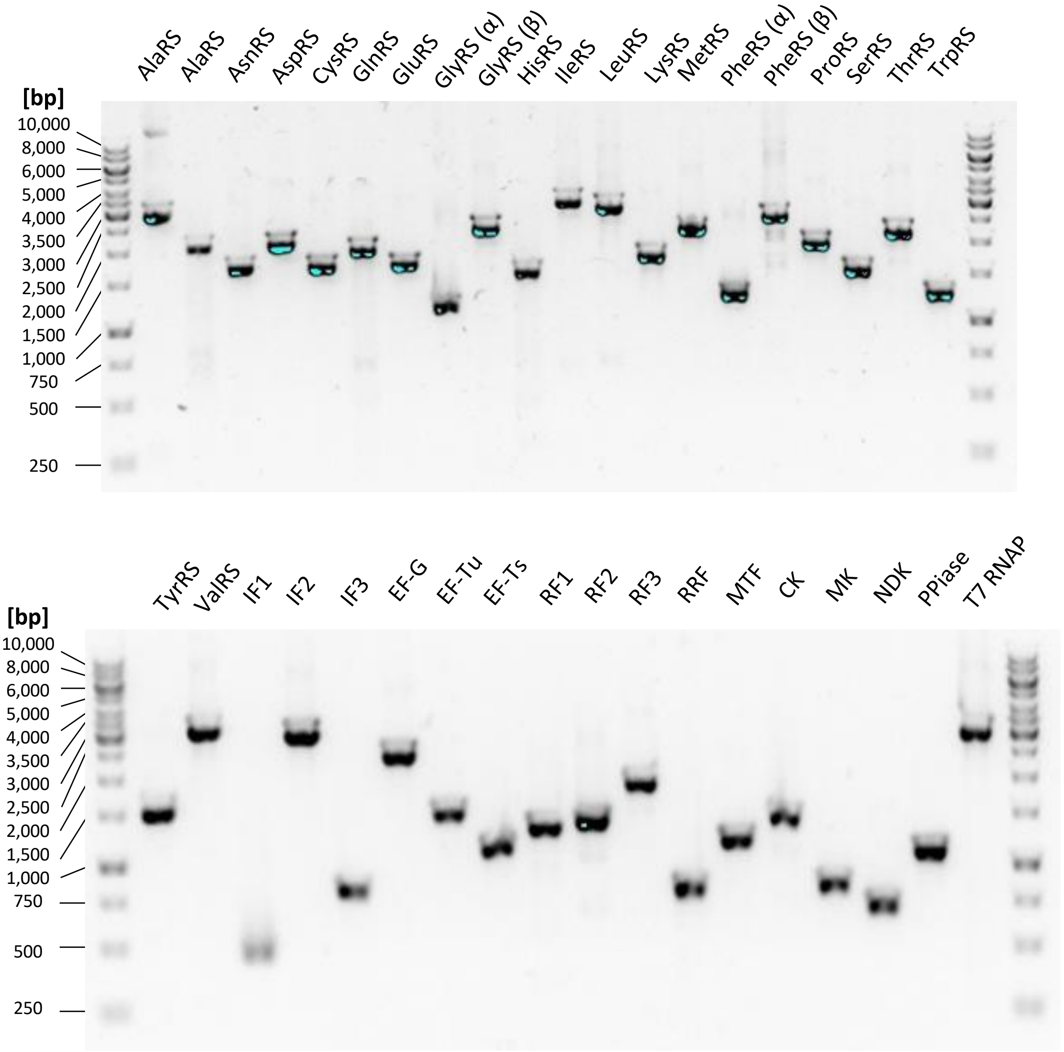

Supplementary Figure 4: **Amplified and purified linear DNA templates encoding each non-ribosome PURE protein run on an agarose gel.** For GlyRS and PheRS, which each consist of two subunits, two linear DNA templates were amplified, with each template encoding one subunit. Full uncropped scans of gels are appended to the end of the Supporting Information.

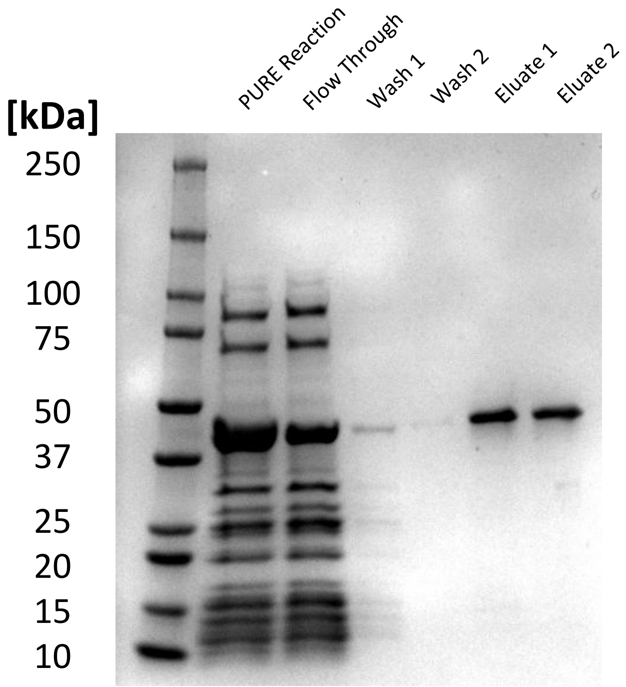

Supplementary Figure 5: **Coomassie-stained SDS–PAGE of EF-Tu purification.** EF-Tu was expressed in a PUREfrex reaction and purified using magnetic beads. Samples from each purification step were run on SDS–PAGE followed by Coomassie staining. Full uncropped scans of gels are appended to the end of the Supporting Information.

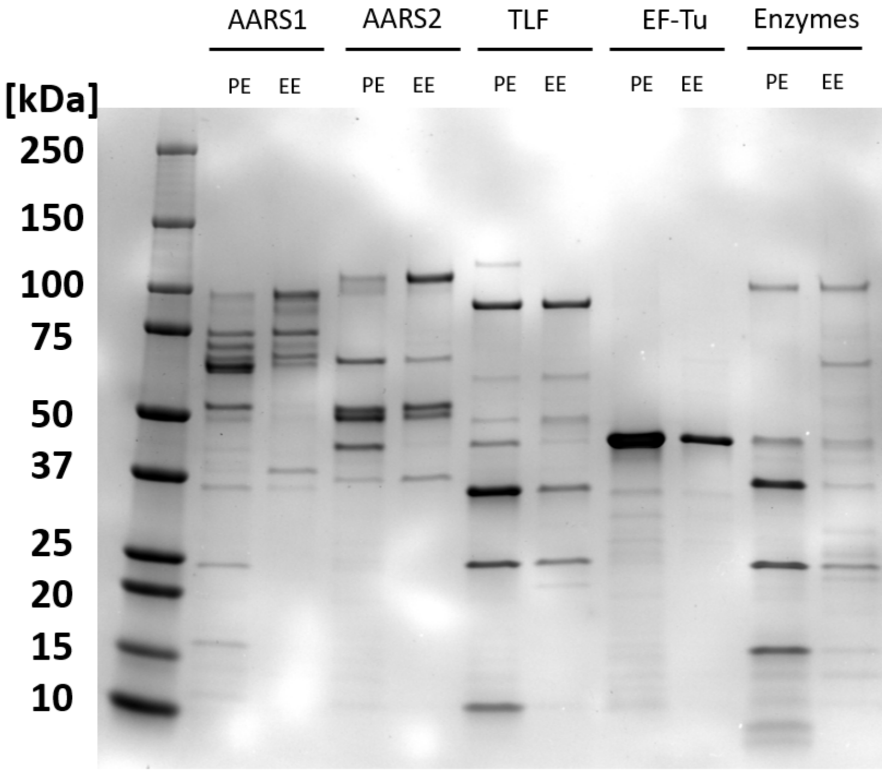

Supplementary Figure 6: **Coomassie-stained SDS-PAGE of final subsets.** Final subsets, which are labeled as PE, were loaded on SDS-PAGE next to their *E. coli* expressed subsets with adjusted concentration, which is labeled as EE, followed by Coomassie staining. In all lanes, 2 µL was loaded, except for EF-Tu, where 1 µL was loaded. EE: *E. coli* expressed, PE: PURE expressed. Full uncropped scans of gels are appended to the end of the Supporting Information.

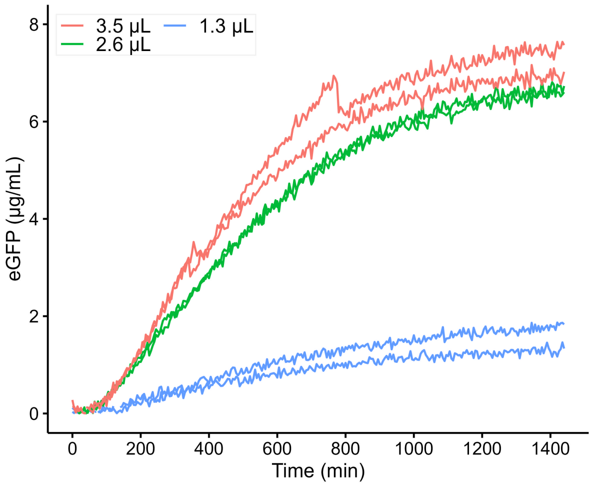

Supplementary Figure 7: **Titration of diluted full PURE in cell-free reactions.** Different volumes of 20-fold–diluted homemade PURE (containing a 20-fold lower concentration of non-ribosome proteins) were added to the other reaction components, including the energy solution, ribosomes, and eGFP template, to determine the optimal amount of diluted non-ribosome PURE protein to supplement the reaction. Fluorescence was measured over time (n=2 for all conditions, technical replicates). Source data are provided as a Source Data file.

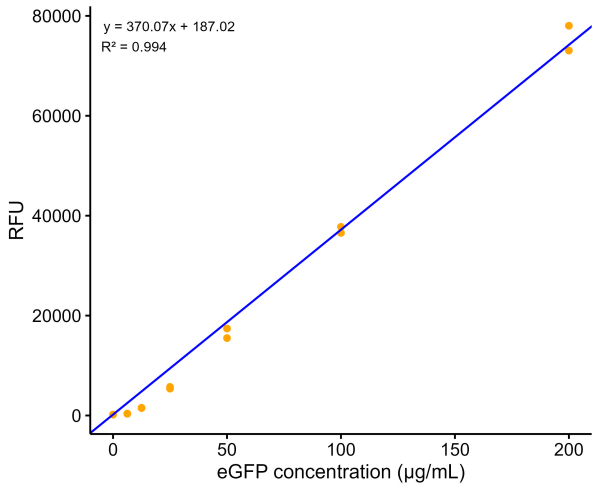

Supplementary Figure 8: **Calibration curve of eGFP.** Different concentrations of recombinant eGFP were prepared in a PUREfrex reaction master mix lacking a DNA template, and their fluorescence was measured with the same settings as the main samples (excitation: 488 nm; emission: 507 nm; gain: 70%). The average value for the second hour was used as the fluorescence signal. The regression curve was constrained to pass through the mean RFU at 0 eGFP concentration. Each concentration was prepared in duplicate (technical replicates). RFU: relative fluorescence units. Source data are provided as a Source Data file.

Supplementary Table 1: **Individual subset functional tests.** Comparison of concentrations of PE subsets used for functional assays in Figure 1c–d and their corresponding concentrations in the original PURE formulation. All concentrations were determined by the Bradford assay (n=2 for all conditions, technical replicates).

**Subset**	**PE subset stock conc.** (µg/mL)	**PE subset conc. in reaction** (µg/mL)	**HM PURE conc. in reaction** (µg/mL)	**HM/PE ratio**
AARS1	410	41	190	4.6
AARS2	460	46	160	3.5
TLF	390	39	210	5.4
EF-Tu	460	46	210	4.6
Enzymes	360	36	58	1.6

Note: PE: PURE expressed, HM: homemade.

Supplementary Table 2: **Regeneration experiment data for proteins from TLF subset.** Summary of PUREfrex reaction volumes, final purified volumes, and the resulting protein concentrations for the functional evaluation of individual proteins from the TLF subset shown in Supplementary Figure 1a. Concentrations were determined by the Bradford assay (n=2 for all conditions, technical replicates).

Protein	Rxn volume	Final volume	Final conc.
IF3	200 µL	40 µL	38 µg/mL
EF-G	100 µL	50 µL	75 µg/mL
EF-Ts	100 µL	50 µL	350 µg/mL

Supplementary Table 3: **Individual protein functional tests.** Functional assay results for specific translation factor components. Comparison of concentrations of PE proteins used for functional assays in Supplementary Figure 1c–d and their corresponding concentrations in the original PURE formulation. All concentrations were determined by the Bradford assay (n=2 for all conditions, technical replicates).

**Protein**	**PE protein stock conc.** (μg/mL)	**PE protein conc. in reaction** (μg/mL)	**HM PURE conc. in reaction** (μg/mL)	**HM/PE ratio**
IF3	38	3.8	19	5.0
EF-G	75	7.5	72	9.6
EF-Ts	350	35	43	1.2

Note: PE: PURE expressed; HM: homemade.

Supplementary Table 4: **Reconstituted PURE functional test.** Comparison of subset and total concentrations for reconstituted PURE (combined PE subsets) used in Figure 2c–d and their corresponding concentrations in the original PURE formulation. All concentrations were determined by the Bradford assay (n=2 for all conditions, technical replicates).

**Subset**	**PE subset stock conc.** (µg/mL)	**Mixing ratio**	**Volume in reaction (%)**	**PE subset conc. in reaction** (µg/mL)	**HM PURE conc. in reaction** (µg/mL)	**HM/PE ratio**
AARS1	410	2.5	2.2	8.9	190	21
AARS2	460	2.5	2.2	10	160	16
TLF	390	10.0	8.7	34	210	6.2
EF-Tu	460	10.0	8.7	40	210	5.3
Enzymes	360	5.0	4.3	16	58	3.6
		**Sum**	26.0	109	830	7.6

Note: PE: PURE expressed, HM: homemade.

Supplementary Table 5: **Single-reaction regenerated PURE functional tests.** Comparison of concentrations of SRR PURE used for functional assays in Figure 2h–i (batch 1) and Supplementary Figure 3b–c (batch 2) and their corresponding concentrations in the original PURE formulation. All concentrations were determined by the Bradford assay (n=2 for all conditions, technical replicates).

<table>
<tr><th>Batch</th><th>SRR PURE stock conc. (µg/mL)</th><th>Volume in reaction (%)</th><th>SRR PURE conc. in reaction (µg/mL)</th><th>HM PURE conc. in reaction (µg/mL)</th><th>HM/PE ratio</th></tr>
<tr><td>1</td><td>700</td><td rowspan="2">26.0</td><td>180</td><td rowspan="2">680</td><td>3.8</td></tr>
<tr><td>2</td><td>410</td><td>110</td><td>6.2</td></tr>
</table>

Note: PE: PURE expressed, SRR: single-reaction regenerated, HM: homemade.

Supplementary Table 6: **DNA sequences.** eGFP linear template, 5' and 3' sequences included upstream and downstream of the coding sequence for all PURE proteins, as well as the primers used for amplification.

**Component**	**Sequence**
eGFP linear template (Red: T7 promoter, Blue: RBS, Green: eGFP coding sequence, Purple: T7 terminator)	gatcttaaggctagagtactaatacgactcactatagggagaccacaacggtttccctctagaaa<br>taattttgtttaacttaagaaggaggaaaaaaaaATGTCTAAAGGTGAAGAATTATT<br>CACTGGTGTTGTCCCAATTTTGGTTGAATTAGATGGTGATGTTAATG<br>GTCACAAATTTTCTGTCTCCGGTGAAGGTGAAGGTGATGCTACTTA<br>CGGTAAATTGACCTTAAAATTTATTTGTACTACTGGTAAATTGCCAGT<br>TCCATGGCCAACCTTAGTCACTACTTTAACTTATGGTGTTCAATGTT<br>TTTCTAGATACCCAGATCATATGAAACAACATGACTTTTTCAAGTCT<br>GCCATGCCAGAAGGTTATGTTCAAGAAAGAACTATTTTTTTCAAAGA<br>TGACGGTAACTACAAGACCAGAGCTGAAGTCAAGTTTGAAGGTGAT<br>ACCTTAGTTAATAGAATCGAATTAAAAGGTATTGATTTTAAAGAAGAT<br>GGTAACATTTTAGGTCACAAATTGGAATACAACTATAACTCTCACAA<br>TGTTTACATCATGGCTGACAAACAAAAGAATGGTATCAAAGTTAACTT<br>CAAAATTAGACACAACATTGAAGATGGTTCTGTTCAATTAGCTGACCA<br>TTATCAACAAAATACTCCAATTGGTGATGGTCCAGTCTTGTTACCAGA<br>CAACCATTACTTATCCACTCAATCTGCCTTATCCAAAGATCCAAACGA<br>AAAGAGAGACCACATGGTCTTGTTAGAATTTGTTACTGCTGCTGGTA<br>TTACCCATGGTATGGATGAATTGTACAAATAAcggctgctaacaaagcccgaaa<br>ggaagctgagttggctgctgccaccgctgagcaataactagcataaccccttggggcctctaaac<br>gggtcttgaggggttttttgctgaaaggaggaactatatcc
Twist forward primer (used for eGFP template amplification)	CAATCCGCCCTCACTACAACCG
Twist reverse primer (used for eGFP template amplification)	TCCCTCATCGACGCCAGAGTAG
5' sequence of PURE templates (Red: T7 promoter, Blue: RBS)	GATCTTAAGGCTAGAGTACTAATACGACTCACTATAGGGGAATTGTG<br>AGCGGATAACAATTCCCCTCTAGAAATAATTTTGTTTAACTTTAAGAA<br>GGAGATATACAT
3' sequence of PURE templates (Purple: T7 terminator)	GATCCGGCTGCTAACAAAGCCCGAAAGGAAGCTGAGTTGGCTGC<br>TGCCACCGCTGAGCAATAACTAGCATAACCCCTTGGGGCCTCTAA<br>ACGGGTCTTGAGGGGTTTTTT
Forward primer for PURE templates	GATCTTAAGGCTAGAGTACTAATACGACTCACTATAGGGGAATTG
Reverse primer for PURE templates	AAAAAACCCCTCAAGACCCGTTTAGAG

Supplementary Table 7: **Protein purification buffers.**

Components	Buffer A	Buffer B	Buffer HT	Stock buffer
HEPES	50 mM	50 mM	50 mM	50 mM
Ammonium chloride	1000 mM	-	-	-
Magnesium chloride	10 mM	10 mM	10 mM	10 mM
Potassium chloride	-	100 mM	100 mM	100 mM
Imidazole (pH=7)	-	500 mM	-	-
Glycerol	-	-	-	30% (v/v)
TCEP	1 mM	1 mM	1 mM	1 mM

Full Uncropped Gel Scans for Supplementary Figures

Original scan for Figure S1b

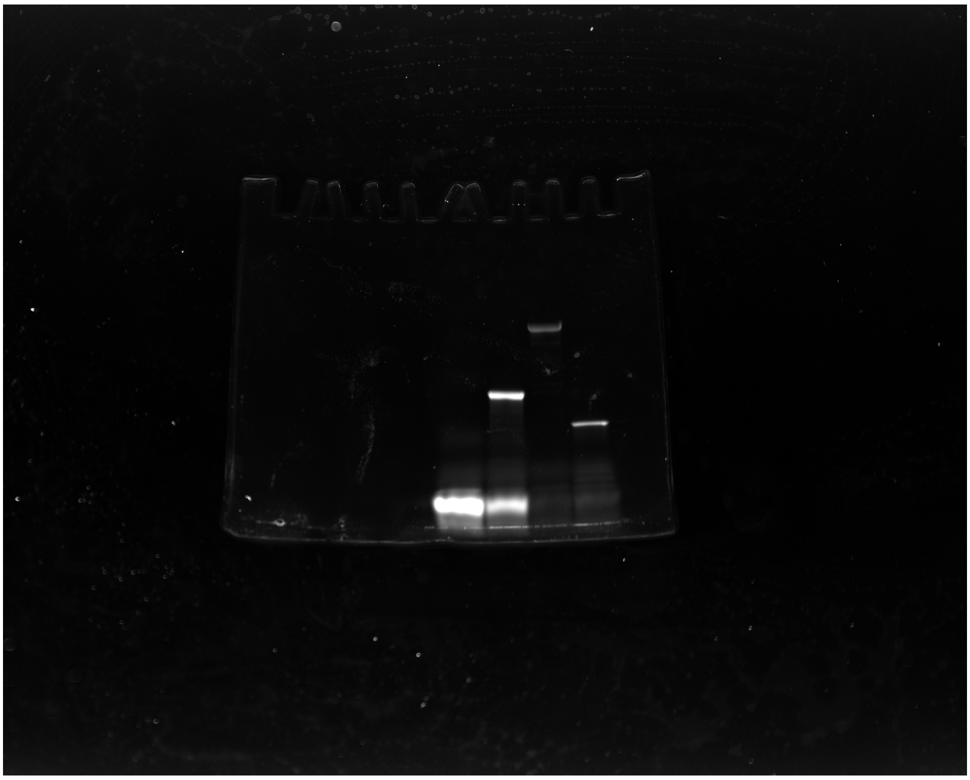

Original scan for Figure S4

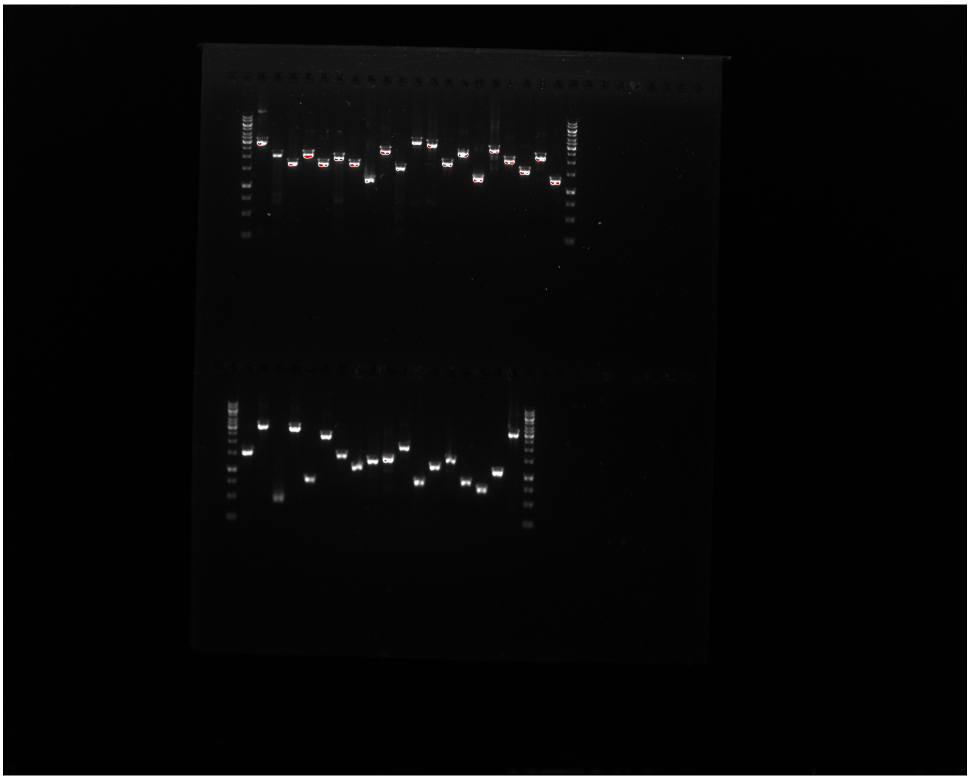

Original scan for Figure S5

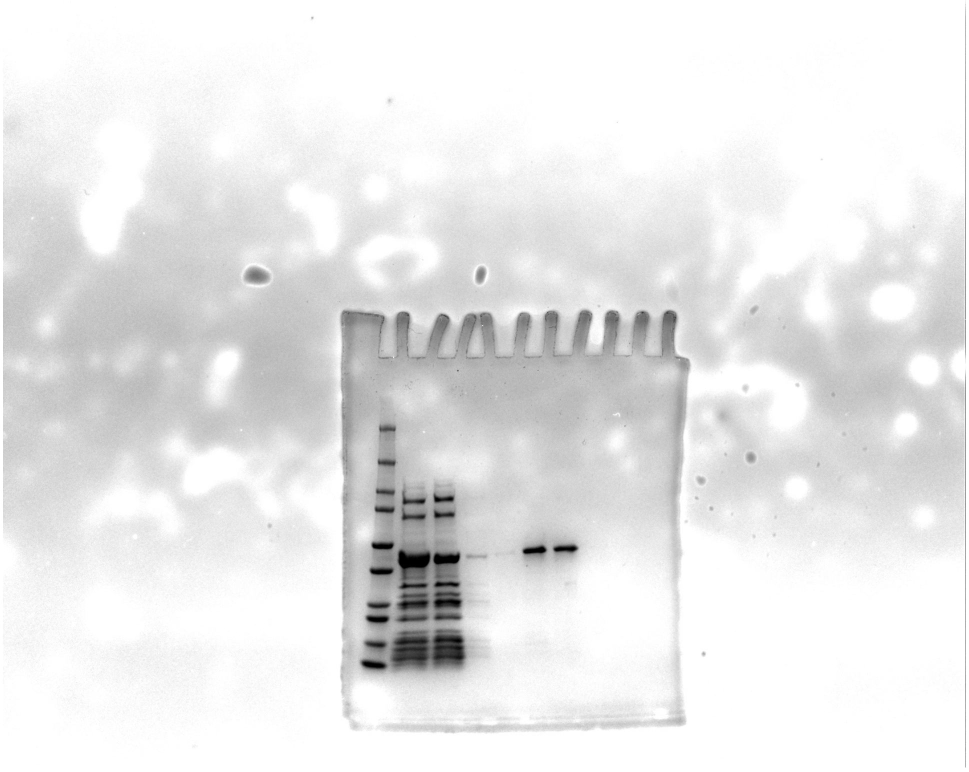

Original scan for Figures 2g and S6

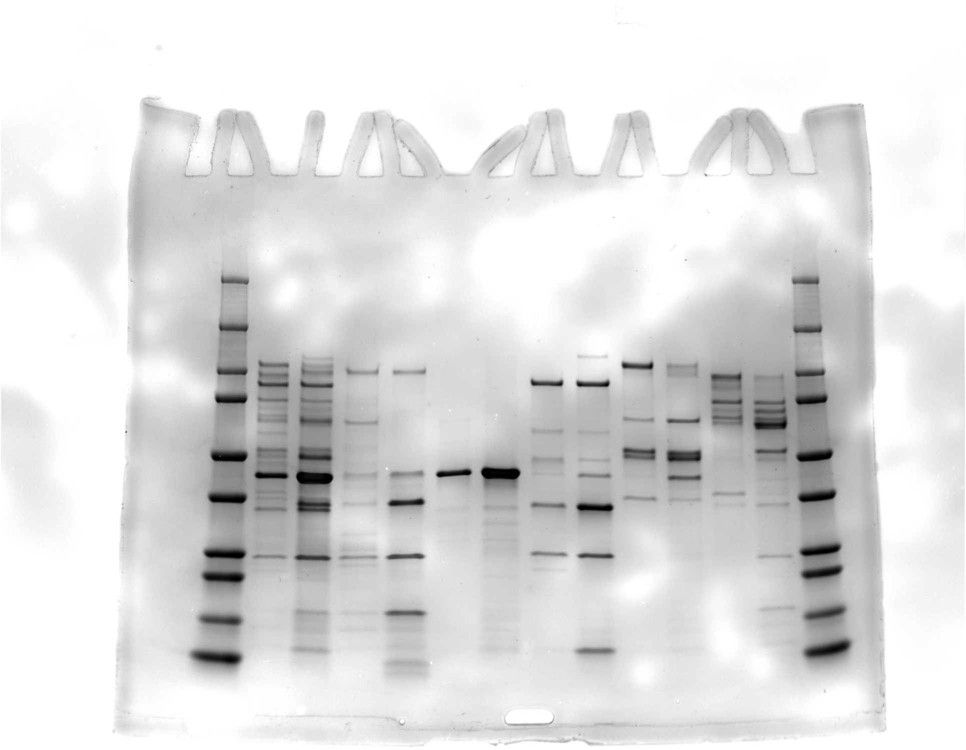

Supplement: Supplementary file 1 — Supplementary Information [file 41467_2026_73337_MOESM1_ESM.pdf]
