# Supplementary material for: PURE makes PURE: reconstitution of the PURE cell-free system from self-synthesized proteins
Source: Nat Commun. 2026 May 22;17:6756. doi: 10.1038/s41467-026-73337-0 (PMC13385756; doi:10.1038/s41467-026-73337-0)
Supplement: Supplementary file 3 — Transparent Peer Review file [file 41467_2026_73337_MOESM3_ESM.pdf]

nature portfolio

Peer Review File

PURE makes PURE: reconstitution of the PURE cell-free system from self-synthesized proteins

Corresponding Author: Professor Sebastian Maerkl

This file contains all reviewer reports in order by version, followed by all author rebuttals in order by version.

Version 0:

Reviewer comments:

Reviewer #1

(Remarks to the Author)
The manuscript entitled "PURE makes PURE: reconstitution of the PURE cell-free system from self-synthesized non-ribosomal proteins," by Mottaghi and Maerkl reported reconstitution of translation system with 36 translation proteins expressed using PURE system. They first demonstrated that each of 36 factors that have been expressed in a PURE system and purified as mixtures were functional. Then performed simultaneous expression of all factors and found that the purified proteins exhibitted a similar level of translation to the diluted original PURE system. I think that the experiments conducted in this study are an important step toward the construction of a self-regenerative reconstituted system and the authors clearly demonstrated significant progress. Also, the experiments and analyses conducted here are technically sound. I have only a few points that need to be commented on by the authors as follows.

1. Lane 51-53. The preprint paper [21] is probably already published in Nat comm at the end of 2025.
2. Fig. S2. Why did the authors amplify two linear DNA template for two subunits of GlyRS, and PheRS? If I remember correctly, these two subunits were designed to be transcribed as an operon in the original expression plasmid.
3. Table S1. Please consider number of significant digits. The data in Table S1 (e.g., "412.5") means that there are four significant figures, but I doubt there are that many in a biochemistry experiment.

Reviewer #2

(Remarks to the Author)
In this manuscript, the authors aim to set a new milestone toward building a universal biochemical constructor based on a cell-free gene expression system, namely the PURE system. The goal is to show that a cell-free gene expression system can self-replicate by producing its own molecular components. In this work, they tackle the issue of non-ribosomal proteins by demonstrating the synthesis of each of the 36 non-ribosomal proteins that make up the PURE system (excluding the proteins and RNA that make the ribosomes). They evaluate their functionality for enabling protein synthesis in ΔPURE reaction. They then demonstrate that the synthesized proteins could be used to regenerate a new functional PURE system, either by pooling the individually expressed and purified ones or by being expressed all at once in a single cell-free reaction. While I believe this work is an important contribution toward engineering a self-replicating biochemical system, the work presented here does not meet publication standards in its present state. The results raise several questions that should be addressed to support the claims. I believe that the manuscript would be improved by addressing the following comments.

1. First, using 'non-ribosomal' is unfortunate and confusing. Non-ribosomal is typically understood as 'peptides or proteins synthesized without ribosomes'. It is easy to change. I suggest changing it or being clear about what it means in the context of the work, as the readers will be confused. In the present work, 'non-ribosomal' proteins mean the proteins (produced by ribosomes) that are not part of the ribosomes' structure.
2. A better characterization of the PURE-generated PURE proteins is required to support the claim that "PURE can make PURE". Their stability and activity over a wide range of time and reaction conditions should be studied to demonstrate that they perform similarly to proteins purified from E. coli.
3. As pointed out by the authors in their introduction, previous works have attempted to synthesize 'non-ribosomal' PURE proteins using the PURE system but failed to obtain the full set and/or showed functionality issues. It seems that in this work, the authors have been able to overcome some of these issues, but the reasons are unclear. The authors should at least

discuss that point and possibly also present data supporting the robustness of their system to ensure the reproducibility of their methods.
4. The manuscript lacks some quantitative aspects. To achieve self-replication, it is important to quantify the concentration of new proteins that can be produced by a given concentration of proteins used in the reaction for each of the 36 proteins expressed in the various conditions presented throughout the manuscript. This correspondence should be made so that we know where we stand in terms of self-reproduction capacity.
5. On the same line, in the single reaction regenerated PURE experiments, the authors detail that their protocol involves using an initial amount of 250 ul PURE reaction to reconstitute a final reaction of 10 ul. Could the authors further discuss this point and propose (and maybe test) optimizations to increase this ratio?
6. Page 3 lines 105-108, on the lack of functionality of TLF and EF-TU subsets, the authors write: “Nonetheless, both of these subsets performed less well than the concentration adjusted control, which is most likely due to slightly reduced functionality of the PURE expressed protein, or possibly due to sub-optimal protein ratios in the case of the TLF subset.” The lack of activities may be the source of low expressions of regenerated PURE, even the ones presented in Figure 2c, and maybe even in 2h. It seems important to further investigate the hypothesis formulated by the authors here to enable further advances with these systems. For the TLF subset, individual proteins may be expressed and purified to study the impact of their respective concentration. The reduced functionality hypothesis should also be tested more extensively.
7. On page 5 lines 137-138 the authors write “we simply assumed equal expression efficiencies for all gene templates”. This hypothesis could be tested to further refine the conditions of the experiment and provide the community with valuable information on self-replicating PURE systems.
8. Would it be possible to further characterize the single-reaction regenerating PURE by expressing the set of proteins at steady state in a chemostat-like setting, as presented in reference 8?
9. Figures 2c 2d 2h graphs are hard to read and must be improved.
10. For each figure, please provide somewhere in the legend the details of the abbreviation used (for figure 1 PE and EE, for figure 2 SRR and Adj.) as right now it is confusing.

Version 1:

Reviewer comments:

Reviewer #1

(Remarks to the Author)
The authors responded my concerns appropriately. Regarding the point 2 (pheRS and glyRS operons), if the authors did not mention the subunit separation in the manuscript, I recommend the authors to describe the reconstruction somewhere in the revised manuscript.

Reviewer #2

(Remarks to the Author)
In the revised version of their manuscript and their responses, the authors addressed most of the issues pointed out in round1. They performed several additional experiments and have provided us with elements to answer most of our concerns.
I think that the manuscript is almost ready for publication. My remaining concern is that, despite the authors' extensive responses, only a few have been included in the manuscript. The discussion section needs to be improved by better discussing the limitations and perspectives of this system. This could be made by adapting the elements presented in sections 3 and 4 of the response file, to expand the discussion on topics such as the poor efficiency of the systems and their improvement perspectives.

Open Access This Peer Review File is licensed under a Creative Commons Attribution 4.0 International License, which permits use, sharing, adaptation, distribution and reproduction in any medium or format, as long as you give appropriate credit to the original author(s) and the source, provide a link to the Creative Commons license, and indicate if changes were made.
In cases where reviewers are anonymous, credit should be given to 'Anonymous Referee' and the source.
The images or other third party material in this Peer Review File are included in the article’s Creative Commons license, unless indicated otherwise in a credit line to the material. If material is not included in the article’s Creative Commons license and your intended use is not permitted by statutory regulation or exceeds the permitted use, you will need to obtain permission directly from the copyright holder.
To view a copy of this license, visit https://creativecommons.org/licenses/by/4.0/

We thank reviewers for their insightful and helpful comments for improving this manuscript. We addressed all of the comments in this rebuttal letter and revised the manuscript accordingly. Responses to all reviewer comments are highlighted in blue.

Reviewer 1

The manuscript entitled "PURE makes PURE: reconstitution of the PURE cell-free system from self-synthesized non-ribosomal proteins," by Mottaghi and Maerkl reported reconstitution of translation system with 36 translation proteins expressed using PURE system. They first demonstrated that each of 36 factors that have been expressed in a PURE system and purified as mixtures were functional. Then performed simultaneous expression of all factors and found that the purified proteins exhibitted a similar level of translation to the diluted original PURE system. I think that the experiments conducted in this study are an important step toward the construction of a self-regenerative reconstituted system and the authors clearly demonstrated significant progress. Also, the experiments and analyses conducted here are technically sound. I have only a few points that need to be commented on by the authors as follows.

We sincerely appreciate the reviewer's positive evaluation of our work and recognition of it as an important step toward the construction of a self-regenerative reconstituted system.

1. Lane 51-53. The preprint paper [21] is probably already published in Nat comm at the end of 2025.

The reference has been updated accordingly.

2. Fig. S2. Why did the authors amplify two linear DNA template for two subunits of GlyRS, and PheRS? If I remember correctly, these two subunits were designed to be transcribed as an operon in the original expression plasmid.

The reviewer is correct that the subunits are organized as an operon in the original expression plasmid. We chose to amplify each subunit separately to provide greater tunability, allowing us to adjust the template ratios for optimized expression. This is particularly important in chemostat settings where expression capacity is limited. However, we ultimately used an equimolar ratio for this specific study.

3. Table S1. Please consider number of significant digits. The data in Table S1 (e.g., "412.5") means that there are four significant figures, but I doubt there are that many in a biochemistry experiment.

We revised the reported values in Figure 1b and Table S1a to ensure that the number of significant figures appropriately reflects the sensitivity and precision of the experimental method.

Reviewer 2

In this manuscript, the authors aim to set a new milestone toward building a universal biochemical constructor based on a cell-free gene expression system, namely the PURE system. The goal is to show that a cell-free gene expression system can self-replicate by producing its own molecular components. In this work, they tackle the issue of non-ribosomal proteins by demonstrating the synthesis of each of the 36 non-ribosomal proteins that make up the PURE system (excluding the proteins and RNA that make the ribosomes). They evaluate their functionality for enabling protein synthesis in ΔPURE reaction. They then demon-

strate that the synthesized proteins could be used to regenerate a new functional PURE system, either by pooling the individually expressed and purified ones or by being expressed all at once in a single cell-free reaction. While I believe this work is an important contribution toward engineering a self-replicating biochemical system, the work presented here does not meet publication standards in its present state. The results raise several questions that should be addressed to support the claims. I believe that the manuscript would be improved by addressing the following comments.

We thank the reviewer for acknowledging that our work is an important contribution toward engineering a self-replicating biochemical system. We have done our best to carefully address all comments and revised the manuscript accordingly.

1. First, using 'non-ribosomal' is unfortunate and confusing. Non-ribosomal is typically understood as 'peptides or proteins synthesized without ribosomes'. It is easy to change. I suggest changing it or being clear about what it means in the context of the work, as the readers will be confused. In the present work, 'non-ribosomal' proteins mean the proteins (produced by ribosomes) that are not part of the ribosomes' structure.

We thank the reviewer for this comment. In previous work we were urged to use the term ”non-ribosomal” in order to more clearly distinguish PURE proteins and ribosomal proteins. But we agree that this unfortunately is a source of confusion. We revised the terminology throughout the manuscript and now use “non-ribosome proteins” to refer to proteins that are translated by ribosomes but are not structural components of the ribosome.

2. A better characterization of the PURE-generated PURE proteins is required to support the claim that “PURE can make PURE”. Their stability and activity over a wide range of time and reaction conditions should be studied to demonstrate that they perform similarly to proteins purified from E. coli.

PURE reactions are typically prepared and run under standard and very well-controlled conditions. The PURE system has a limited reaction lifespan, and longer incubation times do not improve the reaction yield, as the reaction stops after a few hours (Figure 1d) due to the consumption of small-molecule components [1]. The goal of this study was to demonstrate that PURE is capable of making its own (non-ribosome) proteins. In this regard, it is thus not essential that proteins synthesized by PURE function similarly well as E. coli expressed and purified proteins. In the future it certainly will make sense to dive deeper into the functionality of each individual protein, compare it to the gold standard (E. coli expression and purification) and attempt to improve functionality for proteins where it is sub-optimal.

3. As pointed out by the authors in their introduction, previous works have attempted to synthesize 'non-ribosomal' PURE proteins using the PURE system but failed to obtain the full set and/or showed functionality issues. It seems that in this work, the authors have been able to overcome some of these issues, but the reasons are unclear. The authors should at least discuss that point and possibly also present data supporting the robustness of their system to ensure the reproducibility of their methods.

In our previous work we demonstrated continuous sustained self-regeneration of some of the PURE proteins under steady-state conditions on a microfluidic chemostat. Running PURE reactions on a chemostat involves constant dilution of the reaction, and thus removal of self-regenerated proteins from the reaction. Under these conditions, we estimated that the PURE

synthesis rate is currently the major limiting factor (about one order of magnitude too low for full self-regeneration of all non-ribosome proteins).
Here we performed synthesis in batch reactions in which proteins simply accumulate until the reaction stops, followed by purification and up-concentration. This allowed us to answer the question of whether PURE is capable of functionally synthesizing all 36 non-ribosome proteins and is able to make sufficient amounts of each protein in order to reconstitute a second-generation PURE reaction. This is an important pre-requisite for attempting to perform sustained self-regeneration of all non-ribosome proteins on a microfluidic chemostat and achieve steady state.
Incidentally, using this different approach we could confirm the estimate that the synthesis capacity of the PURE system is currently about an order of magnitude too low for sustained self-regeneration (non-degenerative self-regeneration). This directly relates to the next question below.
Some previous studies failed to produce certain PURE proteins in a soluble and functional form [2]. However, recent studies have also managed to express some of the previously problematic PURE components in PURE [3, 4].
Regarding reproducibility, using our method, we were able to produce all pooled subsets functionally and then showed that the single-reaction regenerated PURE system is also functional. Therefore, the results we obtained are consistent. To further demonstrate reproducibility, we repeated the single-reaction regeneration of PURE. The additional data, now included in Figure S3, demonstrates the reproducibility of the regeneration process. We have updated the text highlighting the reproducibility of the process: ”To assess the reproducibility of the process, the single-reaction regeneration experiment was repeated, confirming the successful regeneration of the PURE system (Figure S3).”

4. The manuscript lacks some quantitative aspects. To achieve self-replication, it is important to quantify the concentration of new proteins that can be produced by a given concentration of proteins used in the reaction for each of the 36 proteins expressed in the various conditions presented throughout the manuscript. This correspondence should be made so that we know where we stand in terms of self-reproduction capacity.

We thank the reviewer for pointing this out. We agree that a quantitative calculation is necessary to estimate how far the system is from sustained self-regeneration. The regeneration efficiency of the single-reaction setup has been calculated and added to the Results section. The added text reads as follows: ”For this single-reaction regeneration setup, we used 12.5 µL of PUREfrex protein mixture in the first-generation reaction. This mixture has a total non-ribosome protein concentration of 28 mg/mL based on the Bradford assay, corresponding to 350 µg of non-ribosome proteins. Following purification and concentration, the final non-ribosome protein concentration obtained from this reaction was 700 µg/mL in a volume of approximately 40 µL, yielding a total of 28 µg of non-ribosome protein. Therefore, the regeneration efficiency of the system is 8%, which aligns with our previous estimates that the synthesis capacity of the original PURE system is at least an order of magnitude lower than what is required for sustained self-regeneration of all non-ribosome proteins [5, 6].”

5. On the same line, in the single reaction regenerated PURE experiments, the authors detail that their protocol involves using an initial amount of 250 ul PURE reaction to reconstitute a final reaction of 10 ul. Could the authors further discuss this point and propose (and maybe test) optimizations to increase this ratio?

See above answer. We actually were positively surprised to see that a single-reaction syn-

thesis strategy works at all. We were expecting that smaller pools, or in the worst case scenario one reaction per protein would be required to produce sufficient amounts of protein for a functional second generation PURE reaction. The result that a single-reaction approach was viable is thus already very exciting and encouraging.
For the single-reaction regenerated PURE experiments, we added DNA templates to a 250 µL PUREfrex reaction to synthesize PURE proteins (Figure 2e). After purification, buffer exchange, and concentration adjustment, the final volume of PURE proteins obtained was 40 µL at a concentration of 700 µg/mL. Then for the functional test, we used 2.6 µL of this purified protein mix in a 10 µL PURE reaction. The reason for using 2.6 µL is shown in Figure S7, where higher volumes reduce the yield due to inhibitory effects of the buffer composition. If the initial reaction volume (250 µL) is increased, it would be possible to synthesize more proteins and obtain a final protein mix with a higher concentration, which would likely improve the yield and reaction rate of the second generation reaction. As mentioned in the manuscript, the concentration of regenerated PURE was 3.8× lower than homemade PURE, and we consider this to be the main reason for the lower yield and reaction rate compared to the concentration adjusted control. However, as noted in response to Comment 4, the goal of this study was to demonstrate that a functional PURE system can be reconstituted from proteins expressed by PURE, which we have achieved. This lays the foundation for future work to improve protein synthesis yields and optimize protein ratios.

6. Page 3 lines 105-108, on the lack of functionality of TLF and EF-TU subsets, the authors write: “Nonetheless, both of these subsets performed less well than the concentration adjusted control, which is most likely due to slightly reduced functionality of the PURE expressed protein, or possibly due to sub-optimal protein ratios in the case of the TLF subset.” The lack of activities may be the source of low expressions of regenerated PURE, even the ones presented in Figure 2c, and maybe even in 2h. It seems important to further investigate the hypothesis formulated by the authors here to enable further advances with these systems. For the TLF subset, individual proteins may be expressed and purified to study the impact of their respective concentration. The reduced functionality hypothesis should also be tested more extensively.

Both the TLF and EF-Tu subsets were functional and supported protein synthesis, although at lower efficiency. As the reviewer suggested, the reduced activity of these subsets may contribute to the lower expression rate and yield observed in Figures 2c and 2h, since the other subsets (AARS1, AARS2, and Enzymes) show results comparable to the concentration adjusted control.
The impact of individual protein concentrations on the PURE reaction rate has been extensively characterized in our previous work [5]. In response to the reviewer comment we conducted additional experiments to assess the functionality of the PURE expressed proteins within the TLF subset. We expressed specific proteins known to strongly influence reaction rates, including IF3, EF-G, and EF-Ts [5]. Our results demonstrate that their functionality is comparable to their *E. coli* expressed counterparts (Figure S2). Therefore, the lower rate observed for the TLF subset relative to the concentration adjusted control is highly likely due to sub-optimal protein ratios; specifically, critical proteins within this subset (e.g., IF3, EF-G, and EF-Ts) may be present at lower relative concentrations than in the control.
We updated the text based on the new result: ”In the case of EF-Tu, this is likely due to reduced functionality of the PURE expressed protein. For the TLF subset, the reduced activity is likely attributable to sub-optimal protein ratios, as individually expressed essential proteins from this subset, including IF3, EF-G, and EF-Ts [5], demonstrate functionality comparable to their *E. coli* expressed counterparts (Figure S2)”

7. On page 5 lines 137-138 the authors write "we simply assumed equal expression efficiencies for all gene templates". This hypothesis could be tested to further refine the conditions of the experiment and provide the community with valuable information on self-replicating PURE systems.

For sustained regeneration in a microfluidic chemostat, where the regeneration rate of each protein must match the dilution rate, the DNA template concentration for each protein would likely need to be individually optimized. However, as mentioned in response to Comment 4, the goal of this study was to demonstrate that a functional PURE system can be reconstituted from PURE-expressed proteins. Therefore, precise optimization of the expression efficiency of each individual protein is beyond the scope of the present work and would likely require a different approach in order to not lead to astronomical reagent costs.

8. Would it be possible to further characterize the single-reaction regenerating PURE by expressing the set of proteins at steady state in a chemostat-like setting, as presented in reference 8?

This is indeed the next logical step but outside the scope of this manuscript.

9. Figures 2c 2d 2h graphs are hard to read and must be improved.

Figures 2c, 2d, and 2h have been improved and enlarged to enhance readability.

10. For each figure, please provide somewhere in the legend the details of the abbreviation used (for figure 1 PE and EE, for figure 2 SRR and Adj.) as right now it is confusing.

The abbreviations (PE, EE, SRR, and Adj.) have now been defined in the respective figure legends to improve clarity.

References

[1] Roset Julia, L., Grasemann, L., Stellacci, F. & Maerkl, S. J. Long-term protein synthesis with pure in a mesoscale dialysis system. *ACS Synthetic Biology* **14**, 290–295 (2025).

[2] Wei, E. & Endy, D. Experimental tests of functional molecular regeneration via a standard framework for coordinating synthetic cell building. *BioRxiv* 2021–03 (2021).

[3] Hagino, K., Masuda, K., Shimizu, Y. & Ichihashi, N. Sustainable regeneration of 20 aminoacyl-trna synthetases in a reconstituted system toward self-synthesizing artificial systems. *Science Advances* **11**, eadt6269 (2025).

[4] Schwarz-Schilling, M. *et al.* Autonomous biogenesis of all thirty proteins of the escherichia coli translation machinery. *Nature Communications* (2025).

[5] Ganesh, R. B. & Maerkl, S. J. Towards self-regeneration: Exploring the limits of protein synthesis in the protein synthesis using recombinant elements (pure) cell-free transcription–translation system. *ACS Synthetic Biology* **13**, 2555–2566 (2024).

[6] Lavickova, B., Laohakunakorn, N. & Maerkl, S. J. A partially self-regenerating synthetic cell. *Nature Communications* **11**, 6340 (2020).

We thank reviewers for their additional insightful and helpful comments for improving this manuscript. We addressed the comments in this rebuttal letter and revised the manuscript accordingly. Responses to all reviewer comments are highlighted in blue.

Reviewer 1

The authors responded my concerns appropriately. Regarding the point 2 (pheRS and glyRS operons), if the authors did not mention the subunit separation in the manuscript, I recommend the authors to describe the reconstruction somewhere in the revised manuscript.

The separation of the DNA template encoding each subunit has been added to the Methods section of the manuscript as follows: "For GlyRS and PheRS, which consist of two subunits, we generated separate linear templates for each subunit to allow for ratio optimization, as opposed to the original version amplified from *E. coli*, in which they were transcribed as an operon [1]."

Reviewer 2

In the revised version of their manuscript and their responses, the authors addressed most of the issues pointed out in round1. They performed several additional experiments and have provided us with elements to answer most of our concerns. I think that the manuscript is almost ready for publication. My remaining concern is that, despite the authors' extensive responses, only a few have been included in the manuscript. The discussion section needs to be improved by better discussing the limitations and perspectives of this system. This could be made by adapting the elements presented in sections 3 and 4 of the response file, to expand the discussion on topics such as the poor efficiency of the systems and their improvement perspectives.

The following has been added to the Discussion section, highlighting the current limitations and future perspectives of the self-regeneration of PURE non-ribosomal proteins: "This is an important prerequisite for attempting to perform sustained self-regeneration of all non-ribosome proteins, but also highlights the main challenge which is that the synthesis capacity of the PURE system remains approximately one order of magnitude below the threshold required for non-degenerative self-regeneration. Overcoming this limitation will require significant optimization of the PURE system [2], and possibly the use of dialysis [3] or other approaches in order for the PURE system to produce sufficient protein for sustained self-regeneration."

References

[1] Shimizu, Y. *et al.* Cell-free translation reconstituted with purified components. *Nature biotechnology* **19**, 751–755 (2001).

[2] Ganesh, R. B. & Maerkl, S. J. Towards self-regeneration: Exploring the limits of protein synthesis in the protein synthesis using recombinant elements (pure) cell-free transcription–translation system. *ACS Synthetic Biology* **13**, 2555–2566 (2024).

[3] Lavickova, B., Grasemann, L. & Maerkl, S. J. Improved cell-free transcription–translation reactions in microfluidic chemostats augmented with hydrogel membranes for continuous small molecule dialysis. *ACS Synthetic Biology* **11**, 4134–4141 (2022).
